# Effects of Polysaccharide Elicitors from Endophytic *Fusarium oxysporium* Dzf17 on Growth and Diosgenin Production in Cell Suspension Culture of *Dioscorea zingiberensis*

**DOI:** 10.3390/molecules16119003

**Published:** 2011-10-26

**Authors:** Peiqin Li, Yan Mou, Tijiang Shan, Jianmei Xu, Yan Li, Shiqiong Lu, Ligang Zhou

**Affiliations:** Department of Plant Pathology, College of Agronomy and Biotechnology, China Agricultural University, Beijing 100193, China

**Keywords:** polysaccharides, endophytic fungus *Fusarium oxysporium* Dzf17, *Dioscorea zingiberensis*, diosgenin, cell culture

## Abstract

Three polysaccharides, namely exopolysaccharide (EPS), water-extracted mycelial polysaccharide (WPS) and sodium hydroxide-extracted mycelial polysaccharide (SPS), were prepared from the endophytic fungus *Fusarium oxysporium* Dzf17 isolated from the rhizomes of *Dioscorea zingiberensis*. The effects of the time of addition and polysaccharide concentration on the growth and diosgenin accumulation in cell suspension culture of *D. zingiberensis* were studied. Among them, WPS was found to be the most effective polysaccharide. When WPS was added to the medium at 20 mg/L on the 25th day of culture, the cell dry weight was increased 1.34-fold, diosgenin content 2.85-fold, and diosgenin yield 3.83-fold in comparison to those of control. EPS and SPS showed moderate and relatively weak enhancement effects on cell growth and diosgenin accumulation, respectively. The dynamics of cell growth and diosgenin accumulation when WPS was added to the medium at 20 mg/L on the 25th day of culture were investigated, and results showed that dry weight of cells reached a maximum value on day 30 but the maximum diosgenin content was achieved on day 31.

## 1. Introduction

*Dioscorea zingiberensis* C. H. Wright (Dioscoreaceae) is a well known traditional Chinese medicinal herb, indigenous to the south of China [1,2]. The rhizomes have a high content of disogenin, which is an important precursor of semi-synthetic steroids such as corticosteroids, sex hormones (e.g., progesterone) and other steroidal drugs in the pharmaceutical industry [3,4]. However, overexploitation of natural *D. zingiberensis* has led to a rapid decrease of this plant resource and an acute shortage of the intermediate (diosgenin) for pharmaceutical synthesis. Furthermore, agricultural production of *D. zingiberensis* requires 3–4 years from seedling to mature rhizome, during which time plant growth is highly susceptible to a number of environmental factors, as well as occupying large areas of cultivation land. Consequently *D. zingiberensis *cell culture has been regarded as an alternative means for efficient and controllable production of diosgenin [5].

Plant cell culture is a convenient and efficient culture system for plant science and biotechnology research and development. It has shown great advantages as an alternative to the whole plant system for producing bioactive products, which have been used produce valuable medicinal substances commercially [6,7]. However, the low yield of secondary metabolites in plant cell culture has been a bottleneck for commercialization. Consequently there have been numerous attempts to improve the productivity of secondary metabolites in plant cell culture such as medium optimization, cell line selection, cell immobilization, precursor addition, elicitation, and metabolic engineering [8,9]. Among these manipulation techniques, elicitation is a very attractive strategy for increasing the metabolite production in cell culture system, which can lead to increased yields and shortened culture times [10,11,12,13].

Elicitation is the induction of secondary metabolite production by either biotic or abiotic treatments. Nowadays, the use of pathogenic and non-pathogenic fungal preparations and chemicals as elicitors has become one of the most important and successful strategies to improve secondary metabolite production in plant cell culture [14]. Typical examples cultures enhanced in this way include *Catharanthus roseus *cell culture for catharanthine production [15], *Salvia miltiorrhiza *cell culture for tanshinone production [16], *Taxus* sp. cell culture for paclitaxel produciton [17], *Hyoscyamus muticus* cell culture for sesquiterpene production [18], *Panax ginseng* cell culture for saponin production [19], *Morinda elliptica* cell culture for anthraquinone production [20], and *Dioscorea galesttiana* cell culture for diosgenin production [21]. The fungal elicitors mainly consisted of living or autoclaved fungi (*i.e*., mycelia and spores), crude extracts, as well as fungal peptides, proteins and carbohydrates [5,14]. Elicitation studies in fungal cultures have focused mainly on the use of carbohydrates (*i.e*., polysaccharides and oligosaccharides) prepared from fermentation cultures of fungi. The effects of carbohydrates on plant secondary metabolites depend on their composition, degree of polymerization, concentration and addition time [14,22]. Shikonin production of *Lithospermum erythrorhizon* cells was greatly enhanced by acidic polysaccharides (*i.e*., agaropectin and pectic acid) from agar [23]. Polysaccharide k-carrageenan exhibited elicitation effects on the production of secondary metabolites and various growth characters of chickpea and maize plants [24].

Research on plant endophytic fungi has become a hotspot of research activity in recent years, mainly because of the valuable metabolites with multiple biological activities as well as great potential applications in agriculture, medicine and food industry [25,26,27,28]. To the best of our knowledge, there were few reports about the effects of endophytic fungi on the secondary metabolism of their host plants [29,30,31,32]. *Fusarium oxysporum *Dzf17 is an endophytic fungus isolated from the rhizomes of *D. zingiberensis*. The crude oligosaccharide from this fungus was preliminarily observed in our previous study to have enhancement effects on diosgenin production in *D. zingiberensis *cell culture [30]. The purpose of this work was to investigate the effects of three kinds of polysaccharides from endophytic fungus Dzf17, namely exopolysaccharide (EPS), water-extracted mycelial polysaccharide (WPS) and sodium hydroxide-extracted mycelial polysaccharide (SPS), their addition time and their concentrations in the medium on cell growth and diosgenin production in cell suspension culture of *D. zingiberensis*.

## 2. Results and Discussion

### 2.1. Time Courses of Cell Growth and Diosgenin Production

**Figure 1:**
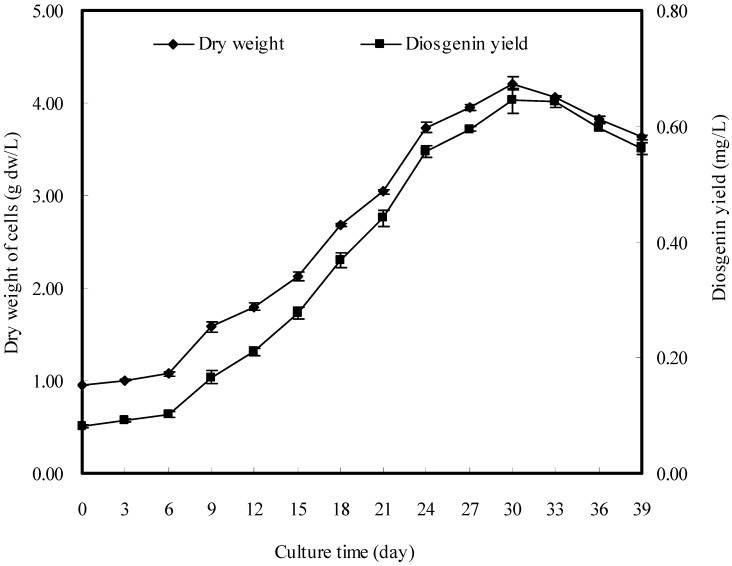
Time courses of cell growth and diosgenin production in cell suspension culture of *D. zingiberensis*.

Time courses of cell growth and disogenin production in cell suspension culture of *D. zingiberensis* without elicitation are shown in Figure 1. The dry weight of cells increased slowly during the first 6-days of culture, and then exhibited a linear increase up to day 30, reaching a highest value of 4.21 g dw/L. Beyond 30 days, cell dry weight did not increase any more but rather decreased slightly. The yield of diosgenin showed the same linear trend as the cell dry weight, but lagging behind the latter. The yield of diosgenin reached its maximum value of 0.644 mg/L on the 30th day after inoculation. Thus, day 30 was determined to be the most suitable time for harvesting *D. zingiberensis* cultured cells to produce diosgenin.

### 2.2. Effects of EPS on Cell Growth and Diosgenin Production

**Figure 2:**
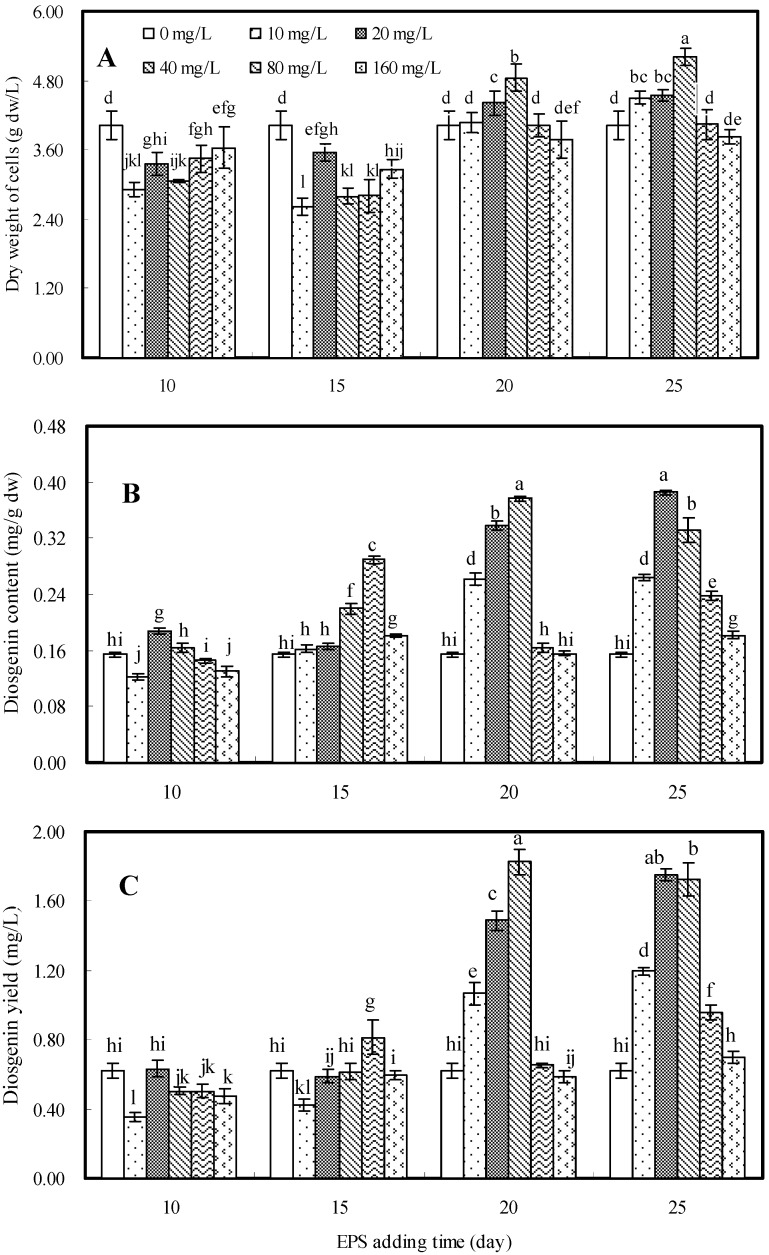
Effects of EPS elicitor on cell growth (**A**), diosgenin content (**B**) and diosgenin yield (**C**) in cell suspension culture of *D. zingiberensis*.

The effects of exopolysaccharide (EPS) as an elicitor on cell growth and diosgenin production in cell suspension culture of *D. zingiberensis* are shown in Figure 2. To investigate the impacts of addition time of EPS in combination with its concentration in medium, elicitation treatment was carried out at different growth stages (days 10, 15, 20 and 25) in cell suspension culture with various concentrations (10 to 160 mg/L) in the medium, and cultures were harvested on the 30th day after inoculation.

Cell growth was inhibited when EPS was added on the either 10th day or 15th day of culture at all tested concentrations, and the dry weight of the treated cell cultures was decreased to 65–91% of control (4.02 g dw/L) (Figure 2A). Obvious enhancing effects on cell growth were observed when EPS was added on the 20th day at concentrations of 20 mg/L or 40 mg/L, and on the 25th day with concentrations of 10 mg/L, 20 mg/L and 40 mg/L, respectively. The maximum dry weight of cells, 5.20 g dw/L, which corresponded to 1.29-fold of control, was obtained when EPS elicitor was added on the 25th day at concentration of 40 mg/L.

Diosgenin yield (mg/L) is the result of the synthesized cell dry weight (g dw/L) and diosgenin content (mg/g dw). The effects of EPS on diosgenin content and yield are shown in Figure 2B and 2C, respectively. When EPS was added either on the 20th day at concentrations of 10 to 40 mg/L or on the 25th day at concentrations of 10 to 80 mg/L, diosgenin yield was obviously improved. The highest diosgenin yield (1.824 mg/L), which was 2.94-fold that of control (0.621 mg/L), was obtained when EPS was added on the 20th day at 40 mg/L.

### 2.3. Effects of WPS on Cell Growth and Diosgenin Production

**Figure 3:**
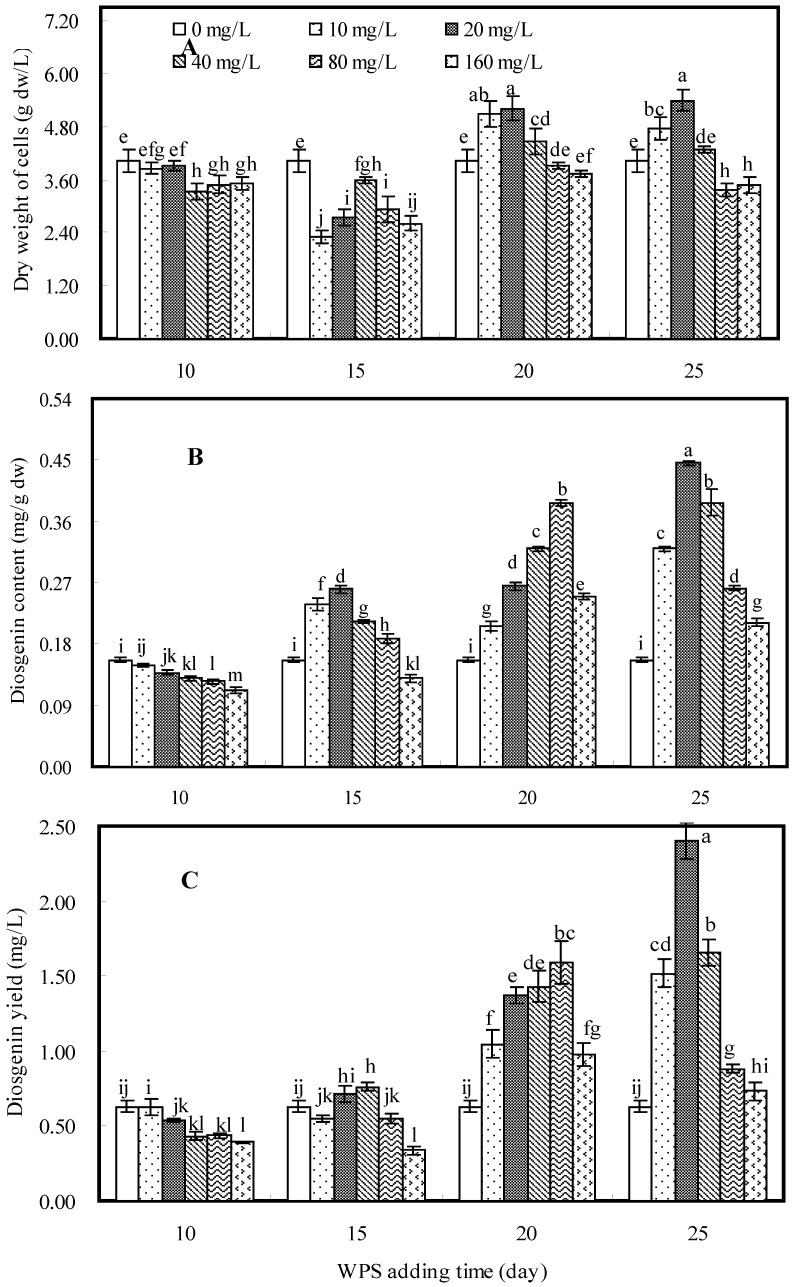
Effects of WPS elicitor on cell growth (**A**), diosgenin content (**B**) and diosgenin yield (**C**) in cell suspension culture of *D. zingiberensis*.

Effects of water-extracted mycelial polysaccharide (WPS) on cell growth and diosgenin production in cell suspension culture of *D. zingiberensis* are shown in Figure 3. A significant increase in cell dry weight was observed when WPS was added as an elicitor either on the 20th day at concentrations of 10, 20 and 40 mg/L, or on the 25th day at concentrations of 10 or 20 mg/L (Figure 3A). When WPS was added on the 25th day at a concentration of 20 mg/L, the maximum cell dry weight value (5.39 g dw/L) was observed, which was 1.34-fold that of control.

The effects of WPS on diosgenin content and yield are shown in Figure 3B and 3C, respectively. When elicitor was added on the 10th or 15th day, it only showed a slight inhibition or weak stimulation effect on diosgenin production. Obvious increasing effects on diosgenin yield were observed when WPS was added on the 20th or 25th day. The highest diosgenin yield of 2.404 mg/L, which was 3.83-fold of control (0.627 mg/L), was observed when WPS was added on the 25th day at a concentration of 20 mg/L.

### 2.4. Effects of SPS on Cell Growth and Diosgenin Production

**Figure 4:**
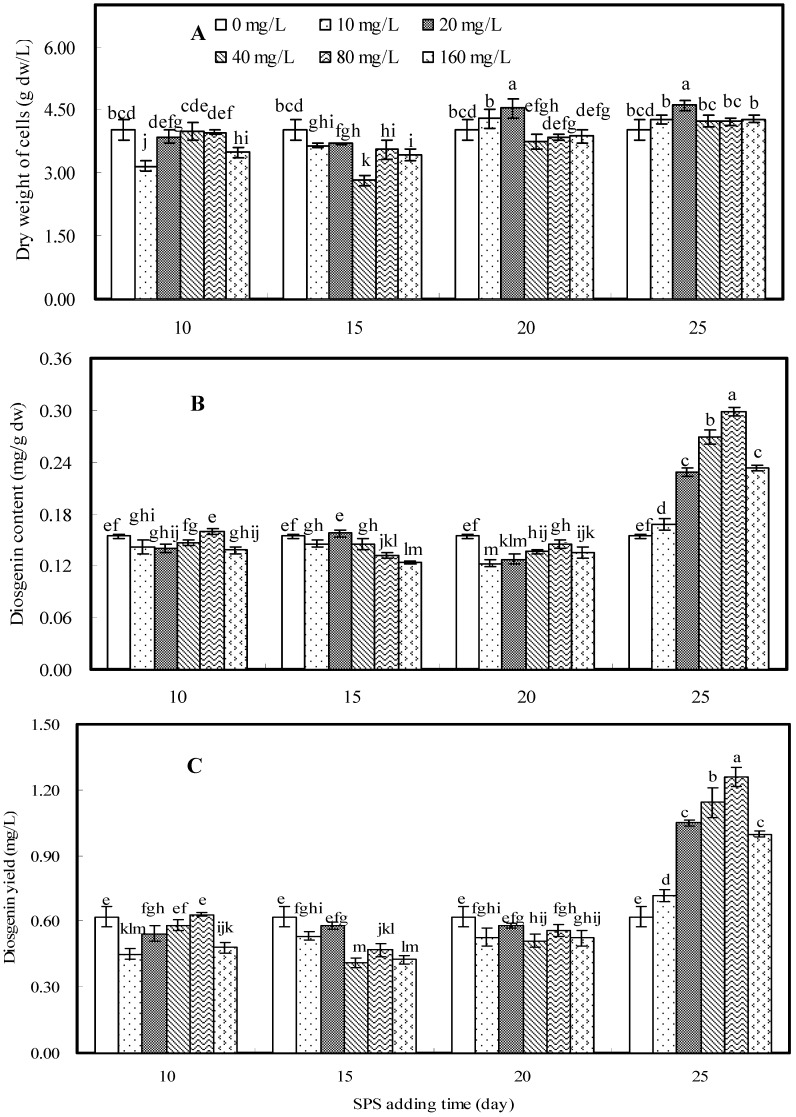
Effects of SPS elicitor on cell growth (**A**), diosgenin content (**B**) and diosgenin yield (**C**) in cell suspension culture of *D. zingiberensis*.

Effects of sodium hydroxide-extracted mycelial polysaccharide (SPS) on cell growth and diosgenin production in cell suspension culture of *D. zingiberensis* are shown in Figure 4. Based on the data depicted in Figure 4A, SPS elicitor had only a slight influence on cell growth. Figure 4B shows the effects of SPS on diosgenin content at different addition times in combination with different concentrations. The highest diosgenin content (0.298 mg/g dw), which was 1.93 fold of control, was observed when SPS was added on the 25th day at a concentration of 80 mg/L. The similar effects of SPS on diosgenin yield are shown in Figure 4C. When SPS was added on the 25th day diosgenin production was obviously stimulated with all tested concentrations. The maximum value of diosgenin yield (1.259 mg/L), which was 2.03-fold of control (0.620 mg/L), was obtained when SPS was added on the 25th day at a concentration of 80 mg/L. When *D. zingiberensis *cells were cultured for 25 days, cell cultures entered into late log phase. As reported in Figure 2, Figure 3 and Figure 4, the best elicitation effects by EPS, WPS and SPS for diosgenin production were observed when polysaccharide elicitor was added on the 25th day in combination with their different concentrations.

### 2.5. Dynamics of Cell Growth and Diosgenin Production after Addition of WPS

**Figure 5:**
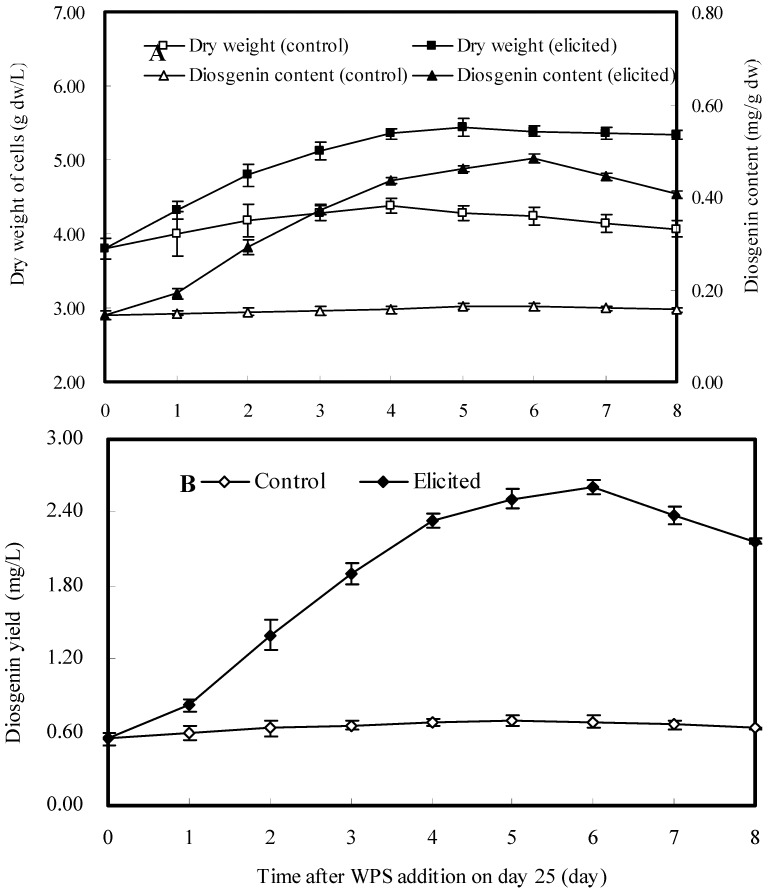
Time courses of cell growth and diosgenin content (**A**) as well as diosgenin yield (**B**) in cell suspension culture of *D. zingiberensis* treated with WPS elicitor (20 mg/L) after 25-days’ continuous culture.

Based on the above elicitation results, among three polysaccharides WPS elicitor showed the best stimulating effects on cell growth and diosgenin production when it was added on the 25th day after inoculation at a concentration of 20 mg/L, so the time courses of cell growth, diosgenin content and diosgenin yield of *D. zingiberensis* suspension cultures treated with WPS elicitor (20 mg/L) on the 25th day were investigated as shown in Figure 5. Cell dry weight showed a moderate linear growth during the first four days, and reached the maximum value five days after treatment (Figure 5A). Six days after treatment, both the diosgenin content (0.453 mg/g dw) and yield (2.606 mg/L) reached their maximum. Then they decreased a little after an additional 2-days co-culture (Figure 5). Thus, an optimum cultivation time for *D. zingiberensis* cells to produce diosgenin by the addition of WPS elicitor to the medium at concentration of 20 mg/L on the 25th day after inoculation was set at 31 days.

Previous reports showed that diosgenin production was enhanced in *Dioscorea floribunda* cell aggregates treated with 2-chloroethylphosphonic acid (2-CEPA) [33]. The autoclaved fungal mycelia of *Alternaria tenuis *at 1.3 g/L in medium were also found to increase diosgenin production in *Dioscorea galeottiana* cell suspension cultures [21]. The polysaccharides from endophytic *F. oxysporium* Dzf17 stimulated diosgenin production in cell suspension culture of *D. zingiberensis *in this study. This indicated that the active components in the fungal mycelia of *A. tenuis* should be the polysaccharides or oligosaccharides which need to be further studied.

## 3. Experimental

### 3.1. Cell Culture

Calli were induced from the root explants of *D. zingiberensis *C. H. Wright as described previously [30], and were subsequently subcultured on Murashige and Skoog (MS) medium supplemented with 6-benzyladenine (1.5 mg/L), naphthaleneacetic acid (1.0 mg/L), agar (8.0 g/L), and sucrose (30 g/L) at an interval of 30 days. The medium pH was adjusted at 5.8 before autoclaving. Each 125-mL Erlenmeyer flask was filled with medium (30 mL), and fresh cells (0.3 g) were inoculated. The flask was then maintained in darkness on a rotary shaker at 120 rpm and 25 °C.

### 3.2. Cultivation of Endophytic Fungus Fusarium Oxysporum Dzf17

Endophytic fungus *Fusarium oxysporum *Dzf17 was isolated from healthy rhizomes of *D. zingiberensis *as reported previously [30]. The mycelia were grown in a 1,000-mL Erlenmeryer flask containing liquid medium (300 mL) consisting of glucose (50 g/L), peptone (13 g/L), NaCl (0.6 g/L), K_2_HPO_4_ (0.6 g/L), and MgSO_4_·7H_2_O (0.2 g/L). All flasks were maintained on a rotary shaker at 150 rpm and 25 °C for 14 days. A total of 150 L of fermentation broth was obtained and centrifuged at 7,741 × *g* for 20 min. The supernatant and mycelia were collected separately. Mycelia were washed twice with deionized water, then lyophilized. About 600 g of mycelia in dry weight (dw) was obtained.

### 3.3. Preparation of Exopolysaccharide

Exopolysaccharide (EPS) was prepared from the supernatant (150 L) mentioned above. Briefly, the supernatant was concentrated under vacuum at 60 °C by a rotary evaporator to a suitable volume and mixed with three volumes of 95% ethanol, then kept at 4 °C for 48 h. After that, the solution was centrifuged at 17,418 × *g* for 15 min, and the precipitate from ethanol dispersion was collected as crude EPS which was further subjected to deproteinization with Sevag reagent (chloroform-*n*-butanol 4:1, v/v), decolorization with H_2_O_2_, and removal of small molecule impurities by dialysis. Polysaccharide mixture with molecular weight greater than 8,000–14,000 Da was kept in dialysis tube. The carbohydrate content of EPS was measured spectrophotometerically by the method of anthrone-sulfuric acid [34], which involved sulfuric acid hydrolysis of the sample in the presence of anthrone agent at 100 °C. The absorbance at 620 nm was measured and calibrated to carbohydrate content using glucose as a reference. After lyophilization, the purified EPS (31.98 g) was stored in a desiccator at room temperature.

### 3.4. Preparation of Water-Extracted Mycelial Polysaccharide and Sodium Hydroxide-Extracted Mycelial Polysaccharide

The lyophilized mycelia (600 g) were powdered in a high power disintegrator, and then subjected to heat circumfluence extraction at 50 °C by 95% ethanol-petroleum ether at 1:1 (v/v) as the refluxing solvent to remove monosaccharide, disaccharide and lipid. The ratio of mycelia powder (g) to refluxing solvent (mL) was 1:5 (w/v). Defatted mycelial powder was obtained by centrifugation (7,741 × *g*, 20 min) and drying in an oven at 40 °C for 2 h, and then immersed in hot water at 90 °C for 2 h with the ratio of water (mL) to the material (g) set at 30:1 (v/w). After that, centrifugation was carried out at 7,741 × *g* for 20 min to separate the residue and the supernatant. The supernatant was condensed to a certain volume under vacuum at 60 °C, and then mixed with three volumes of 95% ethanol, and then kept at 4 °C for 48 h. The following procedure for polysaccharide preparation and purification was the same as the treatments of exopolysaccharide (EPS). The gained polysaccharide (33.24 g) was named as water-extracted mycelial polysaccharide (WPS). The residue not containing WPS was further extracted with 10% sodium hydroxide (NaOH) solution at room temperature for 24 h. The remaining steps were the same described in the treatment of EPS. The obtained polysaccharide (35.89 g) was designated as sodium hydroxide-extracted mycelial polysaccharide (SPS).

### 3.5. Elicitation Treatment of Suspension Cells

Elicitation was carried out with the prepared polysaccharides EPS, WPS and SPS. Stock solutions were prepared by dissolving each polysaccharide in distilled water, and the pH was adjusted at 5.8. The solutions were sterilized by filtering through a microfilter (0.45 μm). The sterilized polysaccharide solutions were stored at 4 °C in a refrigerator before use. Each polysaccharide was added to the medium at the concentrations of 10–160 mg carbohydrate equivalent per liter, respectively. The addition time of each elicitor along with their different concentrations in the medium was studied on days 10, 15, 20 and 25, respectively. The harvest time was determined based on the time courses of cell growth and diosgenin production.

### 3.6. Cell Biomass Determination

The suspension cells were harvested, and separated from the liquid medium by filtration, washed with distilled water to remove residual medium, and then filtrated again under vacuum to obtain fresh cells, which were further lyophilized to a constant dry weight (dw) and expressed as gram dw per liter.

### 3.7. Extraction and Determination of Diosgenin

Diosgenin extraction was carried out as previously described with some modifications [35]. Briefly, powdered dry cultured cells (100 mg) was placed in a tube with 95% ethanol (20 mL), and then subjected to ultrasonic treatment for 1 h. After that, 1 mol/L sulfuric acid (20 mL) was added to each tube, and the contents were hydrolyzed at 121 °C for 2 h. The hydrolysate was extracted three times with petroleum ether. The combined petroleum ether solution was washed twice with 1 mol/L NaOH, and then twice with distilled water. After dehydration with anhydrous sodium sulfate, the petroleum ether solution was then concentrated to dryness under vacuum on a rotary evaporator. The extract was dissolved in acetonitrile, and then filtered through a 0.22 μm filter before analysis.

A high performance liquid chromatography system (Shimadzu, Japan), which consisted of two LC-20AT solvent delivery units, an SIL-20A autosampler, an SPD-M20A photodiode array detector, and CBM-20Alite system controller, was employed. A reversed-phase Agilent TC-C_18_ column (250 mm × 4.6 mm i.d., particle size 5 μm) was used for separation by using a mobile phase of acetonitrile-water (90:10, v/v) at a flow rate of 1 mL/min at 30 °C, and an LCsolution multi-PDA workstation was employed to acquire and process chromatographic data. The injection volume was 20 μL. Changes in absorbance at 203 nm were recorded. The peak area was calibrated to diosgenin content with a chemical standard (Sigma). Diosgenin content in the culture medium was negligible and therefore not determined.

### 3.8. Statistical Analysis

All treatments were performed in triplicate, and the results were represented by their mean values and the standard deviations (SD). The data were submitted to analysis of variance to detect significant differences by PROC ANOVA of SAS version 8.2. The term significant has been used to denote the differences for which *p *≤ 0.05.

## 4. Conclusions

In this work, all the polysaccharides (EPS, WPS and SPS) prepared from endophytic fungus *F. oxysoprium *Dzf17 exhibited obvious effects on cell growth and diosgenin production in cell suspension culture of *D. zingiberensis*. The optimal addition time for the polysaccharide elicitors in *D. zingiberensis *cell suspension culture was suggested to be at late log phase. Among three polysaccharides, WPS showed the most stimulating effects on cell growth and diosgenin production. There is a need to characterize the chemical structures of these polysaccharides from the fungus Dzf17, and to investigate their structure-activity relationships. The crude oligosaccharide prepared by partial acid hydrolysis of *F. oxysporium* Dzf17 fungal cell wall fragments showed a stimulating effect on diosgenin production in *D. zingiberensis *cell culture in our previous study [30]. This indicated that the added polysaccharide in the medium was possibly catabolized into the oligosaccharide fragments which should be the active components to affect the growth and diosgenin production in cell suspension culture of *D. zingiberensis* which need to be further clarified [36]. Other issues including the physiological and ecological roles of these endophytic fungal polysaccharides on host plant cells (*i.e*., cell growth, secondary metabolite biosynthesis), as well as their preparation on a large scale also need to be further studied.
